# Desorption in hemoadsorption therapies: a call for more data

**DOI:** 10.1186/s13054-024-04995-z

**Published:** 2024-06-24

**Authors:** Aron Jansen, Nicole Waalders, Matthijs Kox, Peter Pickkers

**Affiliations:** Department of Intensive Care Medicine, Radboud University Medical Center, Geert Grooteplein Zuid 10, 6500HB Nijmegen, The Netherlands

**Dear Editor,**

We would like to thank Buhlmann et al. for their interest [1] in our study on CytoSorb hemoperfusion treatment during experimental human endotoxemia [2]. The authors describe that, in a patient with Evans syndrome, bilirubin concentrations rapidly declined after initiation of CytoSorb therapy. However, the clearance rate decreased over time and even became negative after 6 h, indicating ‘desorption’ and release of bilirubin back into the circulation. We observed a similar effect for several cytokines in our study, pointing towards saturation of the adsorber. This probably occurs more rapidly if the concentration of the target molecule is higher. Furthermore, as our data demonstrate, saturation does not necessarily occur at the same time for different molecules. Apart from establishing proof-of-principle that treatment with Cytosorb attenuates circulating cytokine concentrations, our study is of further relevance in showing that desorption of cytokines can occur within six hours. We therefore agree with Buhlmann et al. that the *one-size-fits-all* recommendation to exchange the CytoSorb adsorber every eight to 12 h as proposed in the recently published consensus statement on hemoadsorption therapy [3] is too generic, and that a more tailored approach guided by measurement of plasma concentrations might be preferable. In light of the above, the lack of effect on circulating cytokine concentrations demonstrated by a recent meta-analysis [4] may, at least in part, be attributed to desorption, as the adsorber exchange interval was 24 h for the majority of the included studies. We believe that recommendations on adsorber exchange intervals should ideally be derived from larger datasets on clearance, saturation and desorption characteristics for a wider variety of target molecules. This way, clinicians could still make a personalized decision on exchange intervals based on the initial plasma concentration prior to initiation of hemoadsorption therapy without the cost- and labor intensive practice of longitudinal concentration measurements for every individual patient. However, to the best of our knowledge, such datasets are hitherto not available. Therefore, we argue that future clinical trials on efficacy of hemoadsorption therapy should make use of six-hour adsorber exchange intervals in continuous treatment scenarios that are maintained for as long as deemed necessary. In addition, clearance and desorption kinetics should be routinely assessed in these studies to generate the aforementioned much-needed datasets. For use in clinical practice, physicians should be aware that especially patients presenting with extremely elevated plasma concentrations, often observed in early disease stages, might be at risk of desorption within six to eight hours after initiation of hemoadsorption therapy. Therefore, we strongly recommend that the adsorber is exchanged within six hours in these patients.

## Data Availability

Not applicable.
